# A gift for gratitude and cooperative behavior: brain and cognitive effects

**DOI:** 10.1093/scan/nsaa003

**Published:** 2020-01-28

**Authors:** Michela Balconi, Giulia Fronda, Maria Elide Vanutelli

**Affiliations:** 1 Department of Psychology, Catholic University of Milan, Milan 20123, Italy; 2 Research Unit in Social and Affective Neuroscience, Catholic University of Milan, Milan 20123, Italy, and; 3 Department of Philosophy, Università degli Studi di Milano, Milan 20122, Italy

**Keywords:** cooperative task, gratitude, intra-brain activity, gift exchange, near-infrared spectroscopy

## Abstract

Recently, different psychological studies have been interested in identifying the factors that regulate the development and maintenance of long-lasting interpersonal and social relationships. Specifically, the present research explored the link between gift exchange, gratitude and cognitive effects. The behavioral performance and neural activity of 32 participants were recorded during a cooperative game to be played before and after gift exchange. Specifically, participants had to perform the task coupled with a dear friend. Half of the couples were asked to exchange a gift before the task performance; the other half was asked to exchange a gift halfway through the task performance. For hemodynamic brain responses, functional near-infrared spectroscopy was used. Results showed that an increase in cognitive performance occurred after the exchange of gifts, with improved accuracy and lower response times in task performance. Regarding hemodynamic responses, an increase in oxygenated hemoglobin was detected, especially in the dorsolateral prefrontal cortex following the gift exchange. Furthermore, it was observed that gift exchange before the beginning of the task increased the performance level. The present study provides a significant contribution to the identification of those factors that enable the increased cognitive performance based on cooperative relationships.

## Introduction

Psychological research has always been interested in identifying the factors that regulate the development and maintenance of mutual relationships (Cosmides and Tooby, 1996; Hammerstein, 2003; Rand *et al.,* 2009; Schino and Aureli, 2009, 2010). Gift exchange, which has been long represented as a fundamental part of human relations (Mauss, 1954), is a recent area of interest in anthropology, sociology and psychology (Belk, 1976; Banks, 1979; Sherry, 1983; Beatty *et al.,* 1985; Homer and Kahle, 1988). Exchanging gifts, indeed, can be considered an example of commitment and reciprocity that can reinforce social relationships. It represents a sort of symbolic communication (Schwartz, 1967; Belk, 1976; Caplow, 1982) that leads the individuals involved to engage in a social exchange (Burke *et al.,* 2009; Tsvetkova and Macy, 2014) by triggering social learning processes (Bandura, 1977).

Besides being a critical node for the maintenance and development of social relationships, the gift is characterized by a plurality of emotions that represent a key aspect of the experience of giving and receiving (Mick and Demoss, 1990; Belk and Coon, 1993; Mick and Faure, 1998). In fact, emotions can act as social coordination systems (Keltner and Haidt, 1999) that direct individuals goals and motivations (Schwarz, 2007). Between the others, gratitude represents a positive emotion that is often associated with gift exchange (Tesser *et al.,* 1968). It has been defined as a sense of joy that is usually experienced following the receipt of a benefit (Emmons *et al.,* 2004) that is intentionally supplied at a personal cost (Tesser *et al.,* 1968; Gergen *et al.,* 1975; McCullough *et al.,* 2001). Specifically, it has been shown that gratitude facilitates the development and maintenance of mutual direct and indirect relationships (Algoe and Haidt, 2009; DeSteno *et al.,* 2010; Bartlett *et al.,* 2012). Moreover, different research proved that gratitude arouses beneficial effects and positive results in the individuals who experience it, leading to greater life satisfaction, optimism, extraversion and low-stress levels (McCullough *et al.,* 2002). Moreover, it has been shown that gratitude increases moral behavior by stimulating the construction of strong social bonds (McCullough *et al.,* 2001). It is also thought to promote the development of cooperative ties due to the feeling of strong mutuality (Gintis, 2000; Bowles *et al.,* 2001) reciprocal altruism experienced during gift exchange (Boyd and Richerson, 1992; Cosmides and Tooby, 1996; Fehr and Fischbacher, 2003; Gintis *et al.,* 2003; Nowak and Sigmund, 2005).

Cooperative tasks can reflect the human tendency to act jointly that involves helping, sharing and acting prosocially (Vanutelli *et al.,* 2016) and that can influence the immediate and future behavior of the other people involved in the exchange. A large amount of previous studies has shown how cooperation increases shared performance by producing common behavioral effects, such as an improvement in cognitive performance (Balconi and Vanutelli, 2017; Vanutelli *et al.,* 2017; Balconi *et al.,* 2018). More specifically for the context of gift exchange, it has been shown that gratitude can be associated with perceived self-efficacy and some motivational components towards the creation of synergetic actions. This process, indeed, can lead to the achievement of positive results that can induce a performance improvement and is supported by the activation of prefrontal areas (Lukinova and Myagkov, 2016). In particular, the dorsolateral prefrontal cortex (DLPFC) was found to be associated with the implementation of cooperative behaviors (Balconi and Pagani, 2014, 2015), social interactions (Kalbe *et al.,* 2010) and commitment into significant relationships (Petrican and Schimmack, 2008), which are extremely important for efficient interpersonal exchange (Suzuki *et al.,* 2011; Liu *et al.,* 2015; Baker *et al.,* 2016). Previous research has consistently highlighted that the affective, cognitive and behavioral components of social interactions during cooperative actions are supported by specific neural networks connecting the limbic regions and the prefrontal cortex (PFC) (Levitan *et al.,* 2000). A fundamental role in this sense is played by the dorsal (DLPFC) and ventral portions of the lateral PFC, which are mainly involved during cooperative behaviors (Chiao *et al.,* 2009; Balconi and Pagani, 2014, 2015), supporting appropriate action planning (Marsh *et al.,* 2009).

However, despite the evidence of the presence of a relationship between gift exchange, gratitude and cooperation, no previous study has considered all these components at the same time, supporting the effect of these social components towards the cognitive performance. In detail, we thought that a gift exchange, throughout gratitude mechanisms and positive emotions that are experienced during this interaction, could reinforce cooperation and mainly cognitive performance. To answer this research question, we implemented an experimental paradigm where participants coupled in dyads were asked to exchange a gift while performing a cognitive task under conditions of explicit cooperation. Both behavioral and neural responses were recorded for the donor and the recipient by using a functional near-infrared spectroscopy (fNIRS)-based hyperscanning technique. The aim was to investigate if and how the cognitive performance and the brain activity of the participants improved after the gift exchange. In the second instance, we aimed at exploring how the specific moment in which the exchange of the gift took place (at the beginning or in the middle of the interaction) could affect the subsequent responses. At this regard, we hypothesized that an early gift exchange could immediately affect the nature of the relationship, with positive effects on the behavioral performance if compared to a later gift exchange. Furthermore, this study sought to observe the cortical localization related to empathic and cooperative brain areas and rewarding networks related to prosocial behavior. We believed that the frontal areas could be more involved in the case of close, positive interactions as demonstrated by several neuroimaging studies and studies that have shown their involvement in emotional empathic reactivity (Harmer *et al.,* 2001; Rameson and Lieberman, 2009) and cooperative behaviors (Chiao *et al.,* 2009; Balconi and Pagani, 2014, 2015). Thus, the gift exchange effect should improve the self-perception of the degree of cooperation, inducing an increased activation of prefrontal areas finalized to support more cooperative and positive emotional behavior. This brain effect is expected to positively impact on cognitive performance and behavioral performance.

## Methods

### Participants

Thirty-two dyads of female subjects involved in a friendship relationship took part in the present research. All the participants were university students (*M*_age_ = 22.31; s.d._age_ = 1.66). For the recruitment of the participants psychiatric or neurological diseases, the presence of cognitive deficits, clinically relevant stress level and the occurrence of significant stressful life events during the last 6 months were excluded. All the participants took part in the study after signing the informed written consent. The research was conducted in compliance with the principles and guidelines of the Helsinki Declaration and was approved by the local ethics committee of the Department of Psychology of the Catholic University of Milan.

### Procedure

**Fig. 1:**
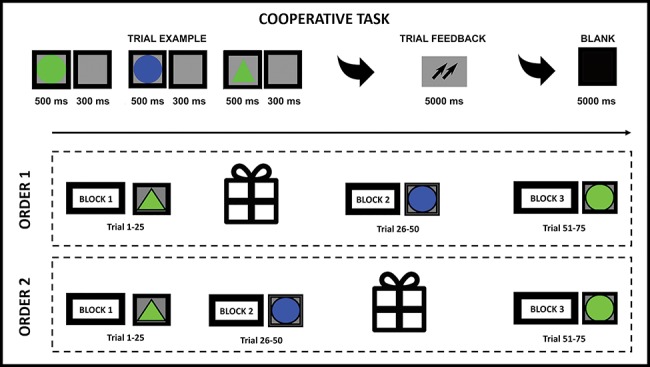
Experimental procedure. Two different procedures were performed: ‘early’ that comprised block 1 (a control condition), gift exchange and then blocks 2 and 3, while ‘late’ comprised block 1, block 2, gift exchange and block 3. Blocks 1, 2 and 3 involve a cooperative task, which consisted of a game of selective attention.

The participants were asked to sit side by side in a low-light room at a distance of 60 cm from two computers and divided by a black screen to avoid eye contact, preventing the couple from the possibility of looking or talking to each other. Specifically, subjects were asked to perform a joint social task that included an exchange of gifts at the beginning or halfway through the task. Specifically, one of the members of each dyad (the donor) was asked to exchange a gift with the partner (the recipient) at the beginning or middle of the activity. The gift delivery was randomized within the dyad: for half of the sixteen pairs (eight dyads), it occurred before the start of the first part of the task (after block 1), while for the other half (eight pairs) at the end of the second block. Specifically, the gift was given from the donor to the receiver face to face. The type of gift was suggested by the experimenter through a panel of gifts: objects, accessories or tickets for visiting a museum or a concert. This way, two different procedures were performed: ‘early’ condition, which comprised the first task block (block 1), gift exchange, and then the second task block (block 2) and the third task block (block 3), while ‘late’ condition that was organized as follows: the first task block (block 1), the second task block (block 2), gift exchange and the third task block (block 3) (see Figure 1). Blocks 1, 2 and 3 involve a cooperative task that consisted of a game of selective attention modified by a previous computerized activity (a single person, Balconi and Pagani, 2015; or of two interacting participants cooperating, (Vanutelli *et al.,* 2016, 2017; Balconi and Vanutelli, 2016a; Balconi *et al.,* 2018); or competing (Balconi *et al.,* 2018)) without gif exchange. In the present version of the task, we opted for the cooperative condition with a specific gift exchange. Specifically, the selective attention task required to memorize a target that was to be subsequently recognized among other different objects by pressing the left/right keys on the keyboard. The target to be memorized could be a circle or a triangle of blue or green color. Each stimulus appeared on the screen for 500 ms with an inter-stimulation interval of 300 ms and an inter-trial interval of 5000 ms duration. The goal of the couples’ members was to be able to synchronize their behavioral responses in terms of speed and accuracy (ACC). At this regard, after the presentation of three stimuli, subjects were given feedback on their degree of cooperation to reinforce their positive outcomes, which was represented by two upward arrows. Participants were instructed that, when appearing on the screen, this symbol indicated the presence of good cooperative strategies. This procedure was adopted in order to reinforce the adoption of joint strategies based on ACC and reaction times (RTs). In fact, by using this feedback, the members of the dyads could implicitly not only learn about their own, but also their friends’ performance and adjust accordingly. Thus, this strategy was meant to improve their awereness and cooperative level.

At the end of the experiment, a questionnaire was given to the participants to investigate the perception of their partner and the game pair during the first and second block tasks, before and after the gift exchange. Specifically, subjects answers to the following open questions: ‘What was the perception of your workmate in the first phase of the game?’, ‘What was the perception of your workmate in the second phase of the game?’, ‘What was the perception of your cooperation in the first phase of the game?’, ‘What was the perception of your cooperation in the second phase of the game?’. Then, participants’ answers have been codified by three expert judges along a Likert scale. Each answer was transformed into a numeric value from 1 to 3 (1 = not in tune/non-cooperative, 2 = in tune/cooperative and 3 = very in tune/very cooperative).

### fNIRS recording and signal processing

**Fig. 2:**
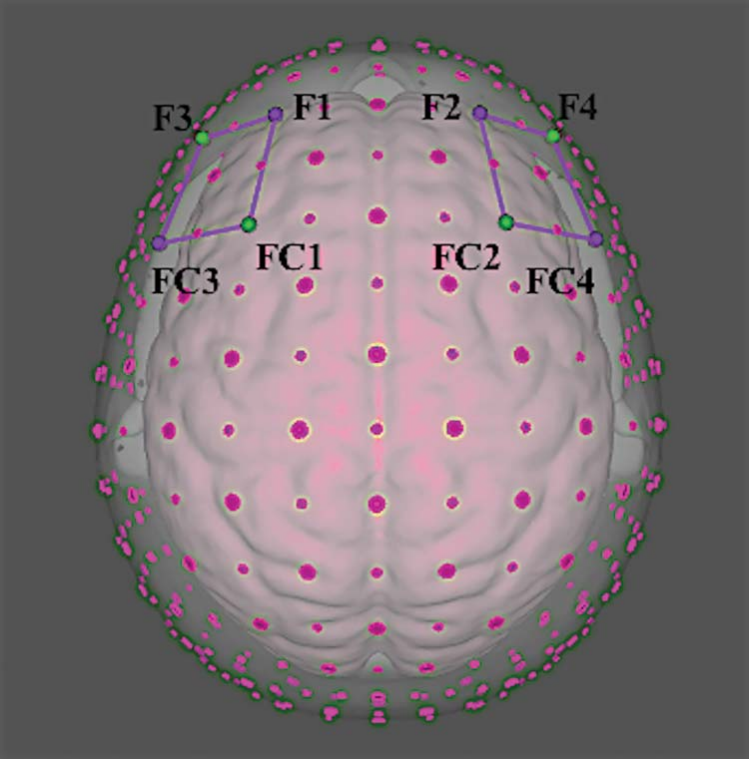
Cortical maps of fNIRS montage. The location of fNIRS optodes: The emitters were placed on FC3–FC4 and F1–F2 positions, while detectors were placed on FC1–FC2 and F3–F4.

For the recording of hemodynamic responses, an NIRScout system was used (NIRx Medical Technologies, LLC, Los Angeles, California) with an 8-optode matrix (four injectors and four detectors) that was placed on the frontal and prefrontal regions of each individual according to the international 10/5 system (Oostenveld and Praamstra, 2001) using an fNIRS cap. For the positioning of the optodes, a distance of 30 mm and a near-infrared light at two wavelengths (760 and 850 nm) were used. In particular, the injectors were positioned concurrently over the positions FC3–FC4 and F1–F2, while the detectors were placed on the following positions: FC1–FC2 and F3–F4 (see Figure 2). In this way, the following channels were acquired: Ch1 (FC3–F3) and Ch3 (FC4–F4) correspond to the left and right, respectively, DLPFC (Brodmann Area 9); Ch2 (FC3–FC1) and Ch4 (FC4–FC2) correspond to the left and right, respectively, dorsal pre-motor cortex (DPMC, Brodmann Area 6); Ch5 (F1–F3) and Ch 7 (F2–F4) corresponding to the left and right,respectively, frontal eye fields (FEF, Brodmann Area 8); Ch6 (F1–FC1) and Ch8 (F2–FC2) correspond to the left and right, respectively, superior frontal gyrus (SFG, Brodmann Area 6). To associate our locations to Brodmann coordinates, we considered sources and detectors’ positions, as well as the area between them, which includes the channel. Then, we looked for the best underlying functional region and the more fitting Brodmann Area. To do so, we combined several references and online atlases (see for example Jurcak *et al.,* 2007; Koessler *et al.,* 2009; Giacometti *et al.,* 2014).

Variations in the concentration of oxygenated (O2Hb) and deoxygenated (HHb) hemoglobin were recorded continuously starting from the acquisition of a preliminary reference test lasting 120 s. After the baseline record, all dyads completed a block of the task prior to gift exchange to familiarise them with the task (block 1). The signals obtained from the eight NIRS channels were acquired with a sampling frequency of 6.25 Hz and analyzed and processed using the nirsLAB software (v2014.05, NIRx Medical Technologies LLC, 15Cherry Lane, Glen Head, NY, USA) based on their wavelength and position, which led to values for changes in the concentration of O2Hb and HHb for each channel. The raw O2Hb and HHb data for each channel were digitally filtered to a filtered band at 0.01–0.3 Hz.

### Data analysis

By using E-prime Software, ACC and RTs were obtained for each subject during the task. ACC was calculated as the percentage of correct responses on the total responses, while RTs were computed starting from stimulus presentation. Then, two mixed-model ANOVAs were applied to ACC and RTs with blocks (1 *vs* 2 *vs* 3) as repeated factor and condition (cond: ‘early’ *vs* ‘late’) and role (role: donor *vs* receiver) as between factors. For all the ANOVA tests, the degrees of freedom were corrected using Greenhouse–Geisser epsilon when appropriate. *Post hoc* comparisons (contrast analyses) were applied to the data. A Bonferroni test was applied for multiple comparisons. In addition, the normality of the data distribution was preliminary tested (kurtosis and asymmetry tests). The normality assumption of the distribution was supported by these preliminary tests.

Two sets of analyses were performed with respect to behavioral (dyadic tuning, perceived cooperation, ACC, RTs) and neurophysiological-dependent measures (fNIRS: O2Hb, HHb measures): ANOVA on behavioral and on neurophysiological measures; trend analysis on behavioral and on neurophysiological measures.

Finally, a correlational analysis (Pearson coefficient) was applied to behavioral and neurophysiological measures, to verify their direct relationship.

### fNIRS analyses

The mean concentration of O2Hb and HHb for each channel was calculated by averaging data across the three blocks, each lasting about 5 min. According to the mean concentrations in the time series, the effect size in every block was calculated for each channel and subject as the difference of the means of the block (m2) and the baseline (m1) divided by the standard deviation (s.d.) of the baseline: *d* = (m2 − m1)/s.d. (Cohen’s *d* value). The procedure was applied to both O2Hb and HHb variations. Although fNIRS raw data were originally relative values and could not be directly compared across subjects or channels, these normalized indices can now be averaged regardless of the unit since the effect size is not affected by the differential pathlength factor (Schroeter *et al.,* 2003; Matsuda and Hiraki, 2006; Shimada and Hiraki, 2006).

Then, four different regions of interest (ROIs) were calculated by averaging left/right homologous channels: the values obtained from Ch1 and Ch3 were averaged as representative of the activity of the DLPFC area, the values obtained from Ch2 and Ch4 were averaged as representative of the activity of DPMC area, the values obtained from Ch5 and Ch7 were averaged as representative of the activity of FEF and the values obtained from Ch6 and Ch8 were averaged as representative of the activity of SFG area. Subsequently, one mixed-model ANOVA was applied to such indices with condition (cond: ‘early’ *vs* ‘late’), blocks (1 *vs* 2 *vs* 3) and ROI (4) as repeated factors and role (role: donor *vs* receiver) as between factor.

This procedure was run following a preliminary step analysis, which also included ‘lateralization’ variable. However, since it was not significant anytime, the variable was then removed from the successive statistical analysis to maintain a higher statistical power.

## Results

### Behavioral data

#### ANOVA

As regards to the questionnaire, two mixed-model ANOVAs were applied to dyadic tuning scoring and perceived cooperation with block (pre *vs* post) as a repeated factor and condition (cond: ‘early’ *vs* ‘late’) and role (role: donor *vs* receiver) as between factors.

Considering dyadic tuning, ANOVA revealed a significant effect for block [*F*(1,28) = 265.15; *P* < 0.0001; *η*_2_ = 0.9], with higher perceived tuning after (*M* = 2.72; s.d. = 0.08) than before (*M* = 1.16; s.d. = 0.06) gift exchange.

With regard to perceived cooperation, ANOVA revealed a significant effect for block [*F*(1,28) = 269.36; *P* < 0.0001; *η*_2_ = 0.91], with higher perceived cooperation after (*M* = 2.75; s.d. = 0.08) than before (*M* = 1.09; s.d. = 0.05) gift exchange.

**Fig. 3:**
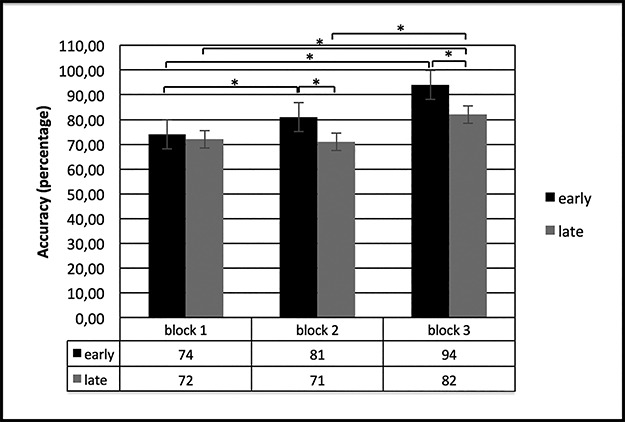
ACC (percentage). The figure shows an increase of performance ACC in ‘early’ than in ‘late’ condition in blocks 2 and 3.

ACC was calculated as the percentage of correct responses on the total responses, while RTs were computed starting from stimulus presentation. For ACC measurement, ANOVA revealed a significant effect for cond [*F*(1,29) = 11.02; *P* < 0.001; *η*_2_ = 0.35], with a better performance (higher percentages) for ‘early’ than ‘late’; block [*F*(2,29) = 9.23; *P* < 0.001; *η*_2_ = 0.32] and cond × block interaction [*F*(2,53) = 7.89; *P* < 0.001; *η*_2_ = 0.29]. Specifically, *post hoc* comparison applied to interaction effect revealed higher ACC in ‘early’ block 2 more than block 1 (baseline) [*F*(1,29) = 11.12; *P* < 0.001; *η*_2_ = 0.33] and in block 3 more than block 1 [*F*(1,29) = 10.90; *P* < 0.001; *η*_2_ = 0.32]. In contrast, ‘late’ showed higher ACC in block 3 more than block 1 [*F*(1,29) = 11.44; *P* < 0.001; *η*_2_ = 0.35] and block 2 [*F*(1,29) = 10.54; *P* < 0.001; *η*_2_ = 0.33]. In addition, comparing ‘early’ *vs* ‘late’ condition, ‘early’ showed increased performance than ‘late’ in block 2 [*F*(1,29) = 11.12; *P* < 0.001; *η*_2_ = 0.33] and in block 3 [*F*(1,29) = 11.87; *P* < 0.001; *η*_2_ = 0.35] (see Figure 3).

**Fig. 4:**
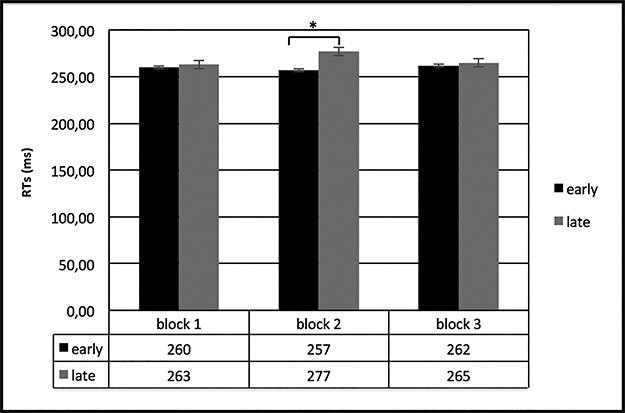
RT responses. The figure shows reduced RTs in ‘early’ than ‘late’ condition in block 2.

Concerning RTs, ANOVA showed a significant effect for cond × block interaction [*F*(1,27) = 15.43, *P* < 0.0001, *η*_2_ = 0.38], with faster RTs in ‘early’ more than ‘late’ in block 2 (Figure 4).

#### Trend analysis

Trend analysis applied to ACC showed significant effect for cond × block interaction [*F*(2,53) = 7.71; *P* < 0.001; *η*_2_ = 0.30]. *Post hoc* comparison applied to interaction effect revealed an increased trend in ‘early’ for ACC related to blocks [with increased ACC for block 1 *vs* block 2 *F*(1,29) = 8.14; *P* < 0.001; *η*_2_ = 0.31; block 1 *vs* block 3 *F*(1,27) = 10.16; *P* < 0.001; *η*_2_ = 0.33; block 2 *vs* block 3 *F*(1,29) = 6.45; *P* < 0.001; *η*_2_ = 0.28]. In contrast, no significant trend effect was found for ‘late’. For RTs, no significant trend effect was found.

### Hemodynamic data

**Fig. 5:**
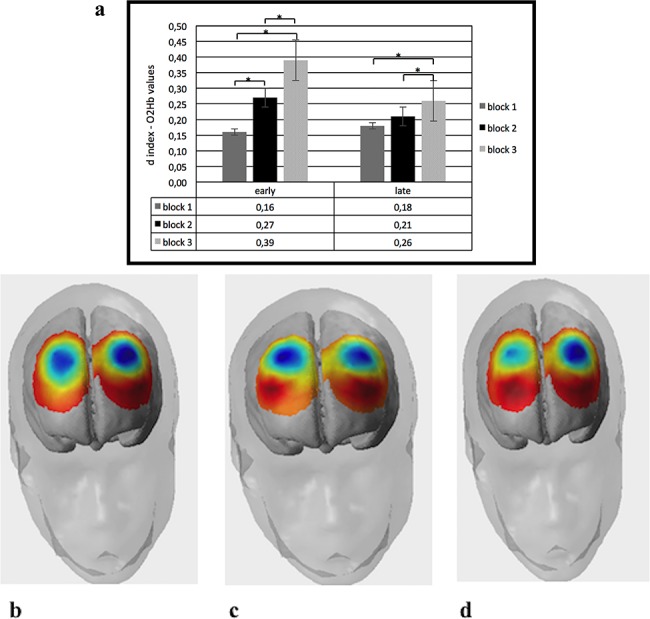
Mean O2Hb values for all recorded channels. (a) The figure shows O2Hb as a function of ‘early’ and ‘late’ conditions. (b) The figure shows the concentration of O2Hb value in DLPFC in block 1 for ‘early’ condition. (c) The figure shows the concentration of O2Hb value in DLPFC in block 2 for ‘early’ condition. (d) The figure shows the concentration of O2Hb value in DLPFC in block 3 for ‘early’ condition.

**Fig. 6:**
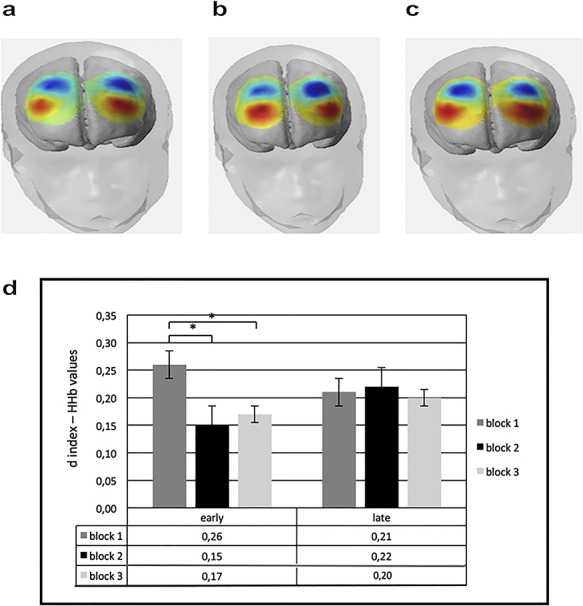
Mean HHb values for all recorded channels. (a) The figure shows the concentration of HHb value in DLPFC in block 1 for ‘early’ condition. (b) The figure shows the concentration of HHb value in DLPFC in block 2 for ‘early’ condition. (c) The figure shows the concentration of HHb value in DLPFC in block 3 for ‘early’ condition. (d) HHb values. The figure shows the concentration of HHb value in DLPFC in the three blocks for ‘early’ and ‘late’ conditions.

*ANOVA*. The statistical analyses were applied to *d* indices for O2Hb and HHb-concentrations. Two mixed-model ANOVAs were applied, respectively, to O2Hb and HHb with condition (cond: ‘early’ *vs* ‘late’), later (lateralization, 1 *vs* 2) blocks (1 *vs* 2 *vs* 3) and ROI (4) as repeated factors and role (role: donor *vs* receiver) as between factor. The ANOVA applied to O2Hb data showed a significant effect for cond [*F*(1,29) = 9.77, *P* < 0.01, *η*_2_ = 0.33], with a general increased brain activity for ‘early’ more than ‘late’. In addition cond × block × ROI interaction effect was significant [*F*(6,72) = 7.99, *P* < 0.01, *η*_2_ = 0.29]. Specifically, as revealed by *post hoc* comparisons, there was an increase of activation in DLPFC area for ‘early’ in blocks 2 and 3 than block 1, respectively, *F*(1,29) = 8.76, *P* < 0.01, *η*_2_ = 0.30; *F*(1,27) = 6.96, *P* < 0.01, *η*_2_ = 0.26]. In addition in ‘early’ block 2 differed from block 3, with higher DLPFC activity in block 3 [*F*(1,29) = 8.45, *P* < 0.01, *η*_2_ = 0.30] (Figure 5a–d). Similarly, in ‘late’ block 3 showed increased DLPFC activity than in blocks 1 and 2, respectively, *F*(1,29) = 7.09, *P* < 0.05, *η*_2_ = 0.27; *F*(1,27) = 7.63, *P* < 0.01, *η*_2_ = 0.26] (Figure 6a–c).

The ANOVA applied to HHb data showed a significant effect for cond [*F*(1,29) = 9.32, *P* < 0.01, *η*_2_ = 0.33], with a general decreased HHb for ‘early’ more than ‘late’. In addition, cond × block × ROI interaction effect was significant [*F*(6,72) = 7.44, *P* < 0.05, *η*_2_ = 0.25]. Specifically, as revealed by *post hoc* comparisons, there was a decrease of HHb in DLPFC area for ‘early’ in blocks 3 and 2 than in block 1, respectively, *F*(1,29) = 6.71, *P* < 0.01, *η*_2_ = 0.26; *F*(1,27) = 6.11, *P* < 0.01, *η*_2_ = 0.26] (Figure 6a–d). No other effect was statistically significant.

*Trend analysis*. Trend analysis applied to O2Hb showed significant effect for cond × block × ROI interaction [*F*(2,72) = 9.05; *P* < 0.001; *η*_2_ = 0.33]. *Post hoc* comparison applied to interaction effect revealed gradually increased values of O2Hb in ‘early’ for block 2 *vs* block 1 [*F*(1,29) = 8.50; *P* < 0.001; *η*_2_ = 0.31]; block 3 *vs* block 1 [*F*(1,29) = 9.13; *P* < 0.001; *η*_2_ = 0.34]; and block 3 *vs* block 2 [*F*(1,29) = 7.09; *P* < 0.001; *η*_2_ = 0.29]. In contrast, no significant trend effect was found for ‘late’. For HHb, no stastistical significant effects were found.

*Correlational analysis*. Pearson coefficients were calculated, respectively, between ACC and RTs and O2Hb and HHb measures. Corrections for multiple comparison (Bonferroni corrections) were applied to the analyses.

Significant direct relationship were found between O2Hb increased DLPFC activity and ACC in block 2 (*r*^2^ = 0.523, *P* ≤ 0.001) and block 3 (*r*^2^ = 0.565, *P* ≤ 0.001) for ‘early’ condition. Similarly, direct relationship were found between O2Hb increased DLPFC activity and ACC in block 3 (*r*^2^ = 0.574, *P* ≤ 0.001) for ‘late’ condition. Finally, significant inverse correlation was found between O2HB increased DLPFC activity and decreased RTs (*r*^2^ = −0.581, *P* ≤ 0.001).

## Discussion

Starting from the evidence of the presence of a relationship among gift exchange, gratitude and cooperation, the present study aimed at investigating if and how gift exchange could influence the behavioral performance as well as the neural activity, through the increased perception of cooperation. Indeed, the main hypothesis was that the emergence of positive emotions arising during this interaction, such as gratitude, could influence the neural and the cognitive responses of the participants by boosting and enhancing the effect of cognitive performance. Then, an experimental joint task was implemented to induce participants, coupled in dyads, to exchange a gift while cooperating each other.

The analyses revealed some significant results: (i) gift donation had positive effects over behavioral responses. (ii) The moment in which the gift was donated could make a difference in influencing the cooperative ties. (iii) The interaction characterized by gift exchange was associated with the involvement of a specific neural network recruiting the frontal areas and in particular the DLPFC. (iv) The effects were detectable for both roles, the donor and the receiver, with a similar effect.

Considering the first main finding, an improvement in the behavioral performance was found after gift exchange with higher ACC rates. This result is in line with what hypothesized concerning the role of gratitude in reinforcing the specific effect of cooperation as it was perceived by the subjects. In fact, previous studies underlined how the presence of a closer interpersonal bond can affect the cognitive level and, therefore, the behavioral responses (Cui *et al.,* 2012; Chung *et al.,* 2015; Balconi and Vanutelli, 2016b; Vanutelli *et al.,* 2017; Balconi *et al.,* 2018). In fact, cooperation implies the ability to adopt common strategies due to some psychological mechanisms, such as perceived self-efficacy, empathy and motivational components that create synergistic actions (Rumble *et al.,* 2010). This effect was also supported by the questionnaire’s results revealing higher perceived tuning and cooperation after the gift exchange.

Interestingly, for both ACC and speed variables, the effect was mediated by the condition factor (II). In fact, the exact timing of gift exchange in the cooperative process did differently influence the behavioral performance. In detail, results showed that when gift donation takes place at the beginning of the social interaction, immediately after the familiarization phase, it can strengthen and maintain the cooperative link, as revealed by faster RTs and more accurate responses in ‘early’ as compared to ‘late’. This effect can be related to the power of the gift to act on strengthening the cooperative bond and on maintaining more substantial and lasting relationships. This phenomenon can be ascribed to the emergence of gratitude and reward mechanisms in mediating the cooperative ties stimulating the construction of social ties (McCullough *et al.,* 2001) and strong reciprocity (Gintis, 2000; Bowles *et al.,* 2001) that influence the social interaction from the beginning, contributing to build a significant bond. A similar pattern emerged in the neural responses. This effect was significantly lower when the gift exchange took place halfway through the task, where the gift has less massive effects. Thus, we can assume that the positive emotions associated with gift donation provided at the beginning of the interpersonal exchange did function as a social glue thanks to reciprocity mechanism. Interestingly, the same principles have been addressed also by the field of economics, where many previous studies explored the importance of reciprocity for efficiency, with generally increased effects of gift receiving over productivity (Fehr *et al.,* 1998; Fehr and Gächter, 1998; Hannan *et al.,* 2002; Charness, 2004).

For the third major finding, it is worth noting that these neural effects were characterized by a specific localized pattern. Specifically, when the gift exchange took place earlier, an increase in oxy levels emerged over the DLPFC. As already discussed, this area is associated with the implementation of cooperative behavior (Balconi and Pagani, 2014, 2015), as well as in bond construction and commitment (Petrican and Schimmack, 2008). However, besides these fundamental functions, the DLPFC appears to be involved in the regulation of the empathic feelings that are involved in the relationship with other individuals (Balconi and Bortolotti, 2012). In our case, empathy appears to be pivotal for the social interaction. In accordance with previous research, indeed, empathy can be considered as the ability to share and understand the emotional state of another individual (de Waal, 2008; De Waal and Suchak, 2010). It can be thought of a multilayer construct that includes different components such as emotional contagion, empathic concern and perspective vision. Subsequently, empathy can be considered as an effective tool for coping with misinterpreted behaviors, thanks to the capacity to take the point of view of another person, to understand and identify with others’ feelings. The mechanism underlying these capabilities can be explained in relation to automatic activation of shared representations in the two inter-agents, which also involves improvement of cognitive abilities (de Waal, 2008) increasing the level of cooperation of the individuals involved in carrying out a shared task. Thus, an empathic framework could, therefore, promote the possibility to maintain and enhance cooperative attitudes (Rumble *et al.,* 2010). Moreover, empathy can promote the implementation of prosocial behaviors. In fact, it functions within a feedback system and can be reinforced by positive, rewarding consequences. By using the provided feedbacks on the screen, the members of the dyads could implicitly learn more about their performance and that of their friends and adjust it accordingly. This way, participants could learn that were the best joint strategies and that, instead, were to be abandoned as inefficient. The focus on the behavioral synchrony in terms of ACC and RT could, thus, promote a reciprocal tuning and trigger a rewarding value over the cooperative exchange.

As revealed by previous literature, in fact, the emotional contagion and the shared representations triggered by empathic attitudes function as positive feedbacks for the promotion of the implementation of future prosocial behavior (Vanutelli *et al.,* 2016). Thus, it appears that expressing gratitude is fundamental for reciprocity and commitment, even if it does not guarantee a useful return benefit. In fact, it signals the importance to maintain beneficial behaviors towards other individuals (Yamamoto *et al.,* 2009).

Once again, such findings are also supported by the questionnaire’s results indicating an increase in both perceived tuning and cooperation. In fact, as demonstrated by previous researches, the experimentation of a pleasant shared experience reinforces the sense of being part of a whole, the sense of perceived self-efficacy and interpersonal cohesion, leading individuals to perceive themselves more in tune with their partner (Cui *et al.,* 2012; Balconi and Pagani, 2015; Chung *et al.,* 2015; Balconi and Vanutelli, 2017).

Finally, it is interesting to underline that the different role of the two actors is generally irrelevant. This means that not only receiving but also (and even more) donating is related to an enhancement of cooperative bonds. This is in line with previous studies (Vanutelli *et al.,* 2016) assessing how acting prosocially without a specific reason or material return could function as a reward *per se* in human beings. However, beside altruistic reasons to act prosocially, other more egoistic mechanisms could be involved, such as the desire to receive attention, or to dissolve the effects of uncomfortable feelings such as guilt (Batson, 2011). Thus, the positive emotions associated with pleasing others can be considered a form of the narcissistic thrust of the subject who has accomplished the benefit. Accordingly, previous literature showed that, when individuals perform a prosocial behavior (such as offering a benefit to someone), the latter can be considered as an instrumental means to obtain personal gain (Batson, 1987). Specifically, even in the absence of obvious external rewards, offering a benefit to another individual involves a form of personal gain that is perceived by the benefactor as a personal reward and self-congratulation (Cialdini and Kenrick, 1976; Bandura, 1977). Starting from these assumptions, more recent perspectives integrated and updated this framework. For example, the extended agency model underlined that narcissism can lead to act more prosocially (Campbell and Foster, 2007) since it would reinforce the rewards experienced from agentic contexts, such as obtaining high status and power. Also, it is thought to substitute the reward coming from more common rewards such as the warmth and connection with others, which is more uncomfortable for the narcissist to experience (Konrath *et al*., 2016).

To conclude, the present study highlights the presence of a strong relationship between gift donation and cooperation, where the role of empathy, positive emotions and rewarding mechanisms can function to reinforce and enhance the social bond, which is visible both at a behavioral and a neural level. Future studies within this line of research could explore more deeply the role of personality factors in mediating these effects, as well as the distinct patterns involved for the two roles: the donor and the receiver. Also, different bond levels could be compared, with strangers, friends and lovers as different groups to investigate if and how reciprocity and sharing a pleasant experience can specifically affect the shared experience. Moreover, considering the different emotional and empathic attitudes in men and women, gender could appear as another variable of interest with regard to the different social role in cooperative dynamics. Furthermore, a larger number of fNIRS channels could be used to investigate how many different cortical areas respond during the implementation of cooperative behaviors. Another point to review in future studies could be the attention task that was used to investigate the cooperation between the two couples. Specifically, in a future study, the latter could be expanded with the addition of different variables for the study of cooperation. Finally, concurrent data could be analyzed to explore the presence of coordinated patterns in the two inter-agents both at a behavioral and a neural level.
